# Edible Alginate–Fungal Chitosan Coatings as Carriers for *Lacticaseibacillus casei* LC03 and Their Impact on Quality Parameters of Strawberries During Cold Storage

**DOI:** 10.3390/foods14020203

**Published:** 2025-01-10

**Authors:** Camila Vilela da Silva Simões, Thayza Christina Montenegro Stamford, Lúcia Raquel Ramos Berger, Alessandra Silva Araújo, José Alberto da Costa Medeiros, Mariane Cajubá de Britto Lira Nogueira, Maria Manuela Estevez Pintado, Silvana Magalhães Salgado, Marcos Antonio Barbosa de Lima

**Affiliations:** 1 Programa de Pós-graduação em Nutrição, Universidade Federal de Pernambuco, Av. Profª Morais Rego, 1235, Cidade Universitária, Recife, PE 50670-901, Brazil; camila.silvasimoes@ufpe.br (C.V.d.S.S.); thayza.stamford@ufpe.br (T.C.M.S.); alessandra.silvaaraujo@ufpe.br (A.S.A.); 2 Laboratório de Microbiologia Aplicada, Centro de Ciências Médicas, Universidade Federal de Pernambuco, Av. Profª Morais Rego, 1235, Cidade Universitária, Recife, PE 50670-901, Brazil; luciaraquelramosberger@gmail.com (L.R.R.B.); alberto_prr@hotmail.com (J.A.d.C.M.); 3 Laboratório de Imunopatologia Keizo-Asami (LIKA), Universidade Federal de Pernambuco (UFPE), Av. Profª Moraes Rego, 1235, Cidade Universitária, Recife, PE 50670-901, Brazil; mariane.lira@ufpe.br; 4 Laboratório de Microbiologia Agrícola e Ambiental, Departamento de Biologia, Universidade Federal Rural de Pernambuco, Rua Dom Manuel de Medeiros, s/n, Dois Irmãos, Recife, PE 52171-900, Brazil; 5 Laboratório de Nanotecnologia, Biotecnologia e Cultura de Células, Centro Acadêmico de Vitória (CAV), Universidade Federal de Pernambuco (UFPE), Rua Alto do Reservatório, s/n, Vitória de Santo Antão, PE 55608-680, Brazil; 6 Centro de Biotecnologia e Química Fina-CBQF, Laboratório Associado, Escola Superior de Biotecnologia, Universidade Católica Portuguesa, Rua Diogo Botelho, 1327, 4169-005 Porto, Portugal; mpintado@porto.ucp.pt; 7 Departamento de Nutrição, Centro de Ciências da Saúde, Universidade Federal de Pernambuco, Av. Profª Morais Rego, 1235, Cidade Universitária, Recife, PE 50670-901, Brazil; silvana.salgado@ufpe.br

**Keywords:** bioactive packaging, biopolymer, fruit, microbial analyses, physiochemical quality, probiotic, self-life

## Abstract

This study investigated the efficacy of an innovative edible coating, composed of fungal chitosan and alginate, functionalized with *Lacticaseibacillus casei* LC03, in both free and microencapsulated forms, to extend the shelf life and enhance the nutritional value of strawberries. *L. casei* LC03 cells were successfully encapsulated in alginate microparticles (MAL) and further coated with chitosan (MALC), resulting in enhanced protection (cell reduction below 1.4 CFU/mL), viability (8.02 log CFU/mL), and encapsulation efficiencies exceeding 90%. The edible coating with *L. casei* microencapsulated in alginate and coated with fungal chitosan (CACLM) significantly improved strawberry preservation by maintaining pH (3.16 ± 0.41), titratable acidity (0.94 ± 0.20), moisture (90.74 ± 0.27), and microbial quality, and delayed the decrease in total phenolic compounds (below 40%) during the storage time of strawberries. While coatings with free *L. casei* (CALF) slightly reduced color parameters (L* value 29.13 ± 2.05), those with chitosan (CACLM) demonstrated lower weight loss (below 6%). Overall, the alginate–chitosan coating, particularly when combined with microencapsulated *L. casei*, proved effective in maintaining the quality, safety, and nutritional value of strawberries during refrigerated storage, highlighting its potential for developing functional, eco-friendly packaging solutions. This research contributes to the development of sustainable food preservation strategies and functional foods.

## 1. Introduction

Strawberries are highly valued worldwide for their appealing sensory qualities and nutritional profile, being rich in vitamins, antioxidants, and dietary fiber [1,2,3]. However, they are among the most perishable fruits due to their high water content and delicate texture, which make them susceptible to rapid microbial decay, moisture loss, and physical deterioration during storage and distribution [1,4,5,6]. This perishability poses a challenge to extending shelf life and maintaining quality. Traditionally, refrigeration has been the main method used to help maintain freshness, but it only partially slows down deterioration processes [1,6,7], with an average shelf life of 5 days at 4 °C [5].

To address the challenges of preserving perishable fruits like strawberries and to meet the growing consumer demand for natural, minimally processed foods with high nutritional value, edible coatings have emerged as promising, eco-friendly technology [6,7,8,9,10]. These coatings align with consumer preferences for natural, minimally processed foods [8,9,11]. By reducing reliance on disposable packaging [7,8,12,13], edible coatings contribute to sustainable practices and support the United Nations Sustainable Development Goals, specifically SDG 2 (Zero Hunger) and SDG 12 (Responsible Consumption and Production) [5,14].

Edible coatings function as semi-permeable barriers that slow moisture loss, reduce respiration rates, and limit oxidative processes, while also helping to prevent microbial contamination on fruit surfaces [5,6,15]. Biopolymer-based coatings, such as those made from chitosan and alginate, are particularly valued for their non-toxic, biodegradable qualities and align well with consumer preferences for clean-label, environmentally friendly preservation methods [12,13,16,17]. Chitosan, produced by the deacetylation of chitin, has been widely studied for its antimicrobial and film-forming capabilities, making it an excellent choice for edible coatings [5,16,18]. Although chitosan is often derived from crustaceans, fungal-sourced chitosan offers a sustainable, hypoallergenic alternative with similar functional properties [15]. Alginate, sourced from brown algae, is another widely used biopolymer in food coatings, prized for its stabilizing and gelling abilities, which strengthen the coating’s structure and enhance its effectiveness in maintaining fruit quality [11,13].

In recent years, incorporating probiotics to functionalize edible coatings has become a promising strategy for enhancing food quality and providing potential health benefits to consumers [2,9,10,12,17]. Furthermore, this technology enables the incorporation of probiotics into non-dairy food matrices, leading to innovative functional foods with unique market appeal [12,19,20]. Among the probiotics, *Lacticaseibacillus casei* LC03 stands out, a well-known strain with health-promoting properties that has demonstrated resistance [21] and beneficial effects across a variety of food matrices, establishing itself as a promising bioprotective agent for fresh produce when incorporated into edible coatings [12,17,22,23]. The probiotic *L. casei* can produce beneficial metabolites such as organic acids and bacteriocins, which can be used as biological preservatives in foods [10]. However, sustaining the viability of the probiotic cells during storage and application to fresh produce continues to be a challenge [22,24]. To address this, microencapsulation—enclosing probiotics in protective materials—has been proposed to improve the stability and effectiveness of probiotics cells within edible coatings [22,24,25].

Microencapsulation has emerged as an effective technique to enhance probiotic stability by shielding cells from environmental stressors, thus aiding in the maintenance of probiotic viability in coatings applied to fresh produce [12,17,26,27,28]. Probiotic microencapsulation is usually performed within a matrix of biopolymeric material, usually a polysaccharide, such as alginate, chitosan, carboxymethylcellulose, and pectin [25]. It is believed that microparticle technology allows the release of probiotic bacteria in concentrations suitable for the intestine, in addition to providing protection against stress generated during food processing and storage, increasing their viability [2,20]. Highlights of edible coatings on fruits, such as strawberries, enriched with free or microencapsulated probiotics, present promising health benefits for consumers.

In this context, the present research aims to evaluate the effectiveness of an edible coating made from fungal chitosan–alginate, functionalized with *L. casei* LC03, in both free and microencapsulated forms, to maintain quality standards and probiotic viability in strawberries during refrigerated storage.

## 2. Materials and Methods

### 2.1. Materials

Fungal chitosan (Chibio^®^, Qingdao, China) was purchased from Chibio Biotech Company (China), showing a degree of deacetylation of 85 ± 5%, and low molar weight (80 ± 10 KDa). The polymer was extracted from non-genetically modified *Aspergillus niger* mycelium. Food-grade sodium alginate was provided by Sigma-Aldrick Brazil Ltd. (São Paulo, Brazil). The other substances were obtained from commercial sources.

The strawberries used were purchased at the Pernambuco Supply Center and transported to the Applied Microbiology Laboratory at the Federal University of Pernambuco, where processing took place. The fruits were selected for the absence of external physiological defects, absence of signs of deterioration caused by microorganisms, weight between 8 and 15 g, and uniform color (>75% of the surface red).

### 2.2. Probiotic Cultivation Conditions

*L. casei* LC03 strain (DSM 27537, PROBIOTICAL^®^, Novara, Italy) was provided by the Department of Food Engineering (UFPE/Recife). *L. casei* LC03 strain was inoculated into 9 mL of De Man, Rogosa, and Shape (MRS) broth (Oxoid, Melbourne, Australia) and incubated at 37 °C for 24 h under anaerobiosis (Anaerobic System Anaerogen, Oxoid, Hampshire, UK). The cells were washed with sterile saline solution (NaCl 0.85% *w*/*v*) three times by centrifugation (6000× *g*, 15 min, 4 °C) and suspended in 5 mL of sterile saline solution for later use, as free cells (control) or encapsulated in calcium alginate microparticles coated with fungal chitosan. The inoculum suspension was standardized by reading optical density (OD) at 660 nm, corresponding to a cell concentration of approximately 9 log Colony-Forming Units per milliliter (CFU/mL).

### 2.3. Encapsulation of L. casei LC03 and Viability During Storage

#### 2.3.1. Ionic Gelation Technique

The microparticles were produced using the ionic gelation technique [28] and designated as alginate microparticles containing *L. casei* LC03 (MAL) and alginate microparticles containing *L. casei* coated with a second layer of fungal chitosan (MALC). Briefly, a cell suspension of *Lactobacillus casei* (10 mL) was mixed with 55 mL of sterile 3% sodium alginate (*w*/*v*) (D3247, AJAX Chemicals Ltd., Sydney, Australia) for 5 min at 2000 rpm using magnetic stirring. This resulted in a cell suspension with an estimated concentration of 10^9^ CFU/mL. Subsequently, the cell–alginate mixture was encapsulated using an encapsulator (B-395 Pro, Buchi, Switzerland). The mixture was loaded into a sterile syringe and extruded through a 300 µm needle (positioned 15 cm from the CaCl_2_ solution) into a 0.5 mol/L sterile calcium chloride (CaCl_2_) solution. The encapsulation process was performed at a vibration frequency of 1500 Hz [28].

The microparticles were then filtered (14 µm) (Whatman Grade 4, GE Healthcare, Chicago, IL, USA), washed, and stored in sterile distilled water, being labeled as MAL. Another sample of these microparticles was immersed in a fungal chitosan solution, diluted in sterile lactic acid (1%) with a pH of 5.7 ± 0.2, adjusted with sodium hydroxide (0.1 mol/L), at a ratio of 1 g of microparticles to 10 mL of chitosan solution. These microparticles were labeled as MALC. In this way, two microcapsules were produced: MAL, covered only with alginate (single layer), and MALC (bilayer), with an additional layer of fungal chitosan (0.5%).

#### 2.3.2. Characterization of Microparticles

The morphology and size of the microparticles were evaluated using optical microscopy via a Leica DM 500 microscope (Leica, Heerbrugg, Switzerland) with an attached Leica ICC50 camera, at 10× magnification. The microstructure of the probiotic microparticles was observed using scanning electron microscopy (SEM).

The microparticles, MAL and MALC, (0.1 g) were lyophilized in cryotubes containing 1 mL of a 10% (*w*/*v*) skimmed milk (Acu-media, Lansing, MI, USA) cryoprotectant solution. Previous experiments have demonstrated that this cryoprotectant effectively preserves the viability of *L. casei* cells during the freezing stage prior to lyophilization. Skimmed milk is a preferred protective agent for bacterial lyophilization, as calcium forms a protective layer around the cell wall, reducing stress during subsequent analysis [22]. Samples were frozen at −80 °C for 24 h, then freeze-dried (Liotop model L-101, São Carlos, Brazil) and retrieved after 48 h [28]. For SEM analysis, the lyophilized microparticles were mounted on aluminum stubs and coated with a thin 10 nm gold layer using the JEOL Smart Coater (DII-29010SCTR, Tokyo, Japan). The samples were then examined using a scanning electron microscope, JEOL JSM 5600 LV (Tokyo, Japan), operating at 25 kV [28].

#### 2.3.3. Encapsulation Efficiency (EE) of *L. casei* LC03

Encapsulation efficiency (EE) was defined as the proportion of viable *L. casei* LC03 cells encapsulated within each microparticle relative to the viable cells present in the initial cell suspension used for microparticle preparation (see Section 2.3.1). To assess EE, 1 g of probiotic microparticles (MAL, MALC) was combined with 9 mL of 0.1M sodium citrate solution and stirred at 1800 rpm for 10 min to disintegrate the microparticles [28]. Following this, a 0.1 mL sample was serially diluted from 10^−1^ to 10^−8^ [22,28]. Then, 100 μL from each dilution was plated, using a microdrop inoculation technique [29], in duplicate on MRS agar and incubated anaerobically at 37 °C for 48 h (Anaerobic System Anaerogen, Oxoid) [5]. Colony counts were recorded and results expressed in log CFU/mL. This method was applied to all subsequent *L. casei* viability assessments. *EE* (%) was calculated using Equation (1), where *N* represents the count of viable encapsulated cells, and *N*_0_ is the count of viable cells prior to encapsulation.

(1) $$EE=\left(N÷N0\right)\times 100$$

#### 2.3.4. Viability of *L. casei* LC03 in Microparticles

*L. casei* cells and encapsulated forms (MAL and MALC) were stored separately in Falcon tubes containing sterile distilled water at 4 ± 1 °C. Cell viability was monitored at intervals of 0, 7, 14, 21, and 28 days. For each time point, 100 μL of standardized free cells (OD 1.8) in distilled water and 1 g of MAL and MALC were used to assess *L. casei* viability, following the methodology outlined in Section 2.3.3 [22,28].

### 2.4. Preparation of Edible Coatings Functionalized with L. casei LC03

The study employed eight distinct treatments to evaluate the impact of various edible coatings and probiotic formulations on strawberries. These treatments included:

- Group 1 (Control): Negative control, sanitized by immersion in 1% sodium hypochlorite.
- Group 2 (Glycerol 1%): Positive control, coated with 1% glycerol solution.
- Group 3 (CA): Coated with 3% alginate solution.
- Group 4 (CH): Coated with 0.5% fungal chitosan solution.
- Group 5 (CALF): Coated with 3% alginate solution containing free cells of *L. casei*.
- Group 6 (CALM): Coated with 3% alginate solution containing MAL (alginate microparticles containing *L. casei* LC03).
- Group 7 (CACLF): Coated with 3% alginate solution containing free cells of *L. casei*, followed by a second layer of 0.5% fungal chitosan.
- Group 8 (CACLM): Coated with 3% alginate solution containing MALC (alginate microparticles containing *L. casei* coated with a second layer of fungal chitosan).

To prepare the glycerol 1% (Group 2) and alginate 3% (Group 3) solutions, 1 mL of glycerol and 3 g of sodium alginate, respectively, were dissolved in 100 mL of sterile distilled water. The solutions were gently stirred at 50 °C until fully homogenized. Glycerol, a common plasticizer in edible coatings [23,30], served as the control in this study (Group 2). This control group aimed to achieve the following: (1) assess the preservation effects of simply covering strawberries with an inert substance, and (2) distinguish between the preservative effects of the inert coating and those attributable to the bioactive compounds present in the other test coatings. Alginate 3% was used to create an edible coating (Group 3), since it is a widely used polymer in edible packaging formulations, and the chosen concentration (3%) aligns with that employed in the microencapsulation process detailed in Section 2.3.3.

A fungal chitosan coating (Group 4) was applied. The chitosan, sourced from Chibio^®^, was used at a sub-inhibitory concentration of 5 mg/mL (0.5%) for *L. casei* (MIC 6–7 mg/mL). Chitosan was dissolved in a 1% lactic acid solution (*v*/*v*). This solution was stirred at 180 rpm for 2 h, and the pH was adjusted to 6 using 1 N NaOH.

The probiotic strain *L. casei* LC03 was incorporated into the edible coatings in two forms: free cells (Groups 5 and 7) and microencapsulated (Groups 6 and 8). A 100 μL aliquot of the free cell suspension or 1 g of probiotic microparticles was added to 10 mL of a 3% (*w*/*v*) sodium alginate solution. This ensured a consistent probiotic concentration of approximately 9 log CFU/mL across all samples. To achieve uniform distribution, the *L. casei* cells and microparticles were mixed with the sodium alginate solution using a magnetic stirrer at 2000 rpm for 5 min.

For experimental Groups 7 (CACLF) and 8 (CACLM), the chitosan solution (5 mg/mL) was applied as a second layer on top of the initial edible coating (CALF and CALM, respectively).

### 2.5. Application of Edible Coating to Strawberry Fruits

To assess the effectiveness of the coatings in preserving strawberry quality, we initially removed the sepals and pedicels. This allowed us to evaluate the coatings’ ability to maintain quality standards even when minor physical damage was present. Then, the strawberries were disinfected in a 1% (*v*/*v*) sodium hypochlorite solution for 15 min, with subsequent washing with sterile water. Following this, they were thoroughly rinsed with sterile distilled water and air-dried at 25 °C for 30 min. For the coating process, strawberries in groups 2–8 were immersed in a sterile edible coating solution for 5 min. After coating, any excess material was allowed to drip off for 2 min, and the samples were then air-dried at room temperature (25 ± 1 °C) for an additional 30 min [30].

Uncoated control samples were treated in a similar manner, but instead of the coating solution, they were dipped in sterile distilled water for 5 min. In groups 7 (CACLF) and 8 (CACLM), strawberries received an additional layer of chitosan gel (5 mg/mL), applied in the same manner as the first layer [30].

**Figure 1:**
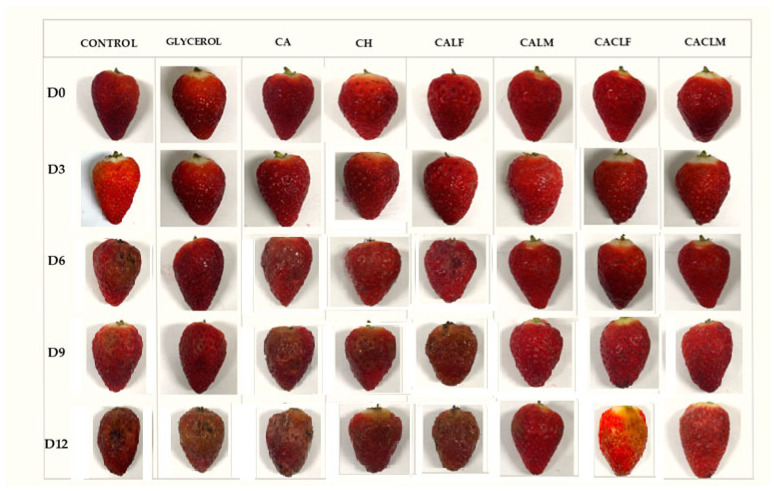
Strawberries without edible coating and with edible coating over 12 days of refrigerated storage (4 ± 1 °C) with the following treatments applied: Control, Glycerol 1% (*v*/*v*), Alginate 3% (CA), Chitosan 0.5% (CH), edible coating with alginate incorporated with *L. casei* LC03 free cells (CALF), edible coating with *L. casei* LCO3 microencapsulated in microparticles of alginate (CALM), edible coating with alginate incorporated with *L. casei* LCO3 free cells with a second layer of 0.5% fungal chitosan (CACLF), edible coating with *L. casei* LCO3 microencapsulated in microparticles of alginate with a second layer of 0.5% fungal chitosan (CACLM).

Each treatment group consisted of 48 strawberries, making a total of 384 strawberries for the experiment. Triplicate analyses were conducted for each time point. The samples were stored under controlled refrigerated conditions at 4 ± 1 °C and 85–90% relative humidity, using a BOD (Biochemical Oxygen Demand) incubator, for 12 days (Figure 1). This storage temperature was chosen based on the methodology outlined by Shahbazi (2018) [30].

### 2.6. Physicochemical Characteristics of Coated and Uncoated Strawberry Fruits

The physicochemical characteristics of coated and uncoated strawberries were analyzed every 3 days (0, 3, 6, 9, 12 days), as per the method set out by Araújo et al. (2024) [5]. These analyses were as follows: soluble solids, pH, titratable acidity, and moisture. The moisture analysis was performed by heating the previously crushed strawberries in an oven at 70 °C for 24 h until dry mass was obtained, and the result was expressed as a percentage. The total soluble solids content determination used a benchtop refractometer (Model AUS JENA, Jena, Germany) and the results were presented as a percentage of soluble solids. The pH was measured using a digital pHmeter (Model: Micronal B474, São Paulo, SP, Brazil). The titratable acidity, expressed as a percentage of citric acid, was determined by titrating the sample with 0.1 N NaOH solution to the set point at pH 8.2 using a digital pHmeter (Model: Micronal B474, Brazil).

### 2.7. Color Evolution

Color analysis was conducted starting from the time of treatment application (day 0) and continued over a 12-day storage period at 4 ± 1 °C, with assessments taken every three days (days 0, 3, 6, 9, and 12). The color measurements were obtained three times in the equatorial region of each strawberry using the CIELab System (L*, a*, and b*) with a colorimeter (MINOLTA Co., Osaka, Japan). Each strawberry was evaluated at two different positions, recording the brightness (L*), the red–green chromatic coordinate (a*), and the yellow–blue chromatic coordinate (b*). Using these values, chroma (C*), which represents color intensity, and hue angle (*H*°), representing color tone (0° = red-purple, 180° = blue-green), were calculated following Equations (2) and (3) [5,31].

(2) $$H^{o}=\tan^{-1}\left(\frac{b^{*}}{a^{*}}\right)$$

(3) $$C^{*}=\sqrt{{\left(a^{*}\right)}^{2}+{\left(b^{*}\right)}^{2}}$$

### 2.8. Weight Loss

Weight loss of the samples was evaluated during refrigerated storage (4 ± 1 °C) on days 0 and 12, according to the method set out by Melo et al. [31]. All strawberries from each group were weighed individually with a Marte model AY22.0 analytical balance. The calculation for mass loss was performed according to Equation (4) and expressed as a percentage of weight loss to the initial weight. The arithmetic mean of the weight of each strawberry per group was considered for application in the formula [5,31]. Analyses were performed in triplicate.

(4) $$\%PP=\left(PI-PF\right)\times 100\;$$

where:

*PP*: percentage of weight loss in the period.

*PI*: initial mass of the sample on day 0 in grams.

*PF*: final sample mass in grams [5,31].

### 2.9. Microbiological Quality and L. casei LC03 Survival During Strawberry Storage

Strawberry microbiological analysis was conducted from the beginning of treatment application (day 0) over a 12-day storage period at 4 ± 1 °C, with assessments taken at three-day intervals (days 0, 3, 6, 9, and 12). For E. coli/total coliforms, Staphylococcus coagulase positive, total aerobic mesophiles, Salmonella spp., and total yeast and mold, analyses were performed using the 3M™ Petrifilm™ system (3M do Brasil Sumaré/SP). All microbiological analyses were conducted in duplicate.

To assess the viability of *L. casei*, serial dilutions were performed in peptone water up to 10^−8^, while for other microbiological analyses, dilutions were made up to 10^−4^. Strawberries coated with edible coatings containing microencapsulated *L. casei*—namely, Groups 6 (CALM) and 8 (CACLM)—underwent a microparticle-breaking protocol (as described in Section 2.3.3) to release and quantify viable probiotic cells. This step was essential as the microparticles remained stable for up to 28 days in refrigerated, sterile distilled water (Section 2.3.4 and Section 3.3). To determine the quantity of microparticles within the edible coating, the strawberries used for the L. casei viability test were scraped using a sterile scalpel, with approximately 500 ± 20 mg of microparticles per strawberry collected and weighed.

For additional microbiological tests and to quantify free L. casei, 10 g of strawberries were manually homogenized in a sterile bag containing 90 mL of sterile peptone water. Serial dilutions were then prepared up to 10^−4^ (microbial analyses) and to 10^−8^ (*L. casei*, free or microencapsulate) in sterile peptone solution for subsequent plating on 3M™ Petrifilm™ Count Plates. Escherichia coli and coliform quantification (CFU/g) was performed on 3M™ Petrifilm™ *E. coli*/Coliform Count Plates, which contained a modified violet-red bile medium and a glucuronidase activity indicator. After 24 h of incubation at 42 ± 1 °C, E. coli colonies appeared blue, while coliform colonies varied from blue to red.

Quantification (CFU/g) of Staphylococcus coagulase positive was conducted on 3M™ Petrifilm™ STX Staph Express Plates, containing modified Baird Parker chromogenic medium, yielding red-violet colonies after incubation at 37 °C for 24 h. Mesophilic aerobic bacteria were quantified using 3M™ Petrifilm™ Aerobic Count Plates with modified Standard Methods nutrients, where total aerobic bacteria appeared as red colonies after 48 h at 35 °C. Yeast and mold counts were performed on 3M™ Petrifilm™ Yeast and Mold Count Plates, which contain nutrients supplemented with antibiotics, a cold-water-soluble gelling agent, and an indicator that aids yeast and mold enumeration.

For presumptive Salmonella sp. presence, 25 g of strawberry samples were incubated at 41 °C for 24 h in 3M™ Salmonella Enrichment Base Medium with a 3M™ Salmonella Enrichment Supplement, followed by plating on 3M™ Petrifilm™ Salmonella Express (SALX) Plates, which utilize a selective and differential chromogenic medium for Salmonella. After incubation at 41 °C for 48 h, presumptive Salmonella colonies presented as red with yellow zones, occasionally accompanied by gas bubbles.

### 2.10. Total Phenolic Content (TPC)

The total phenolic content (TPC) of strawberries was determined using the Folin–Ciocalteu method [32,33]. Briefly, 10 g of randomly selected strawberries were homogenized in 200 mL of 80% methanol using a blender for 10 min. The homogenate was incubated in a water bath at 40 °C for 2 h and then filtered [32,33].

To determine TPC, 0.5 mL of the filtered sample was mixed with 2.5 mL of 10% Folin–Ciocalteu reagent and 2 mL of 7.5% sodium carbonate. The mixture was vortexed and incubated in a water bath at 50 °C for 5 min. After cooling to room temperature, the absorbance was measured at 765 nm using a BioTek μQuant Biospectro spectrophotometer (Winooski, VT, USA). The TPC was expressed as milligrams of gallic acid equivalents per kilogram of sample (mg GAE/kg) [2].

### 2.11. Statistical Analysis

The data were assessed via descriptive analysis and expressed as average ± standard deviation of two independent experiments (repetitions) in triplicate. Significant differences (*p* < 0.05) were determined by ANOVA and Tukey’s test using the software Origin Pro version 8.5.

## 3. Results and Discussion

### 3.1. Characterization of Microparticles

**Figure 2:**
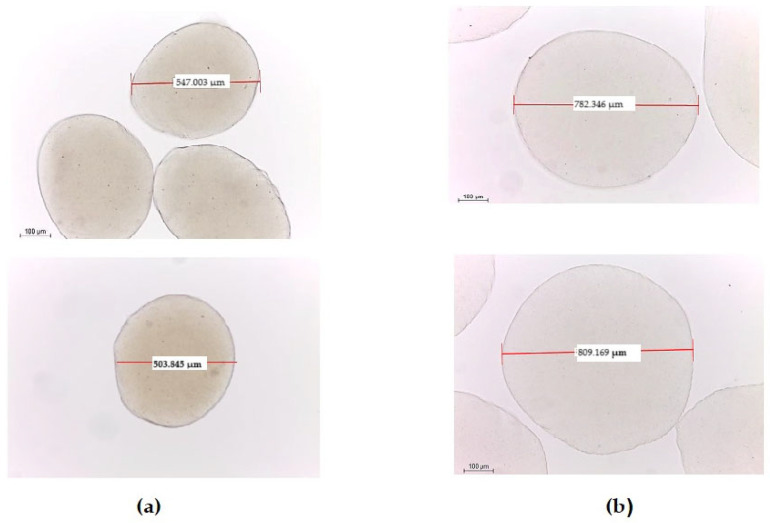
Optical microscopy images of microparticles at 10× magnification: (**a**) microparticles of alginate containing *L. casei* LC03 (MAL) and (**b**) microparticles of alginate containing *L. casei* coated with a second layer of 0.5% fungal chitosan (MALC).

Optical microscopy images demonstrated that both MAL and MALC microparticles were intact, spherical, and uniformly dispersed without signs of agglomeration (Figure 2). The average diameters were 525 ± 29.69 µm for MAL and 795 ± 19 µm for MALC, in line with findings in the literature on alginate–chitosan microcapsules, where particle size can range between 500–800 µm due to variations in coating thickness and cross-linking density [34,35].

**Figure 3:**
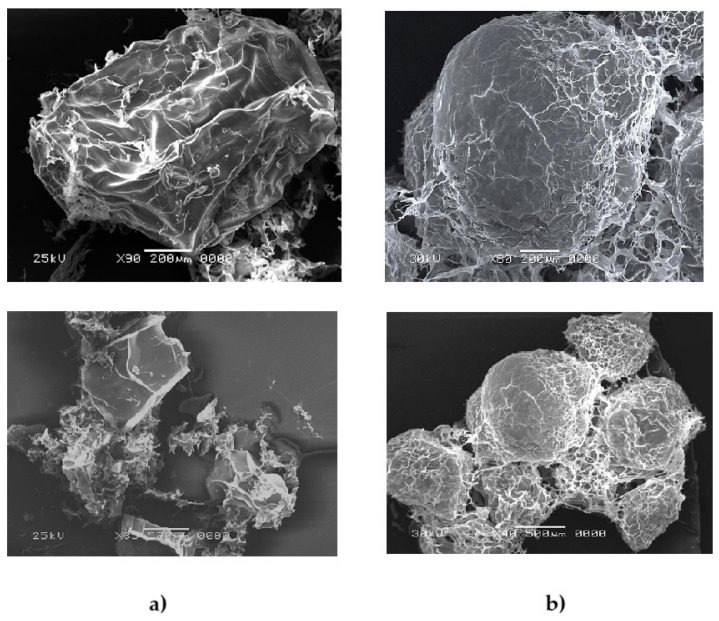
Scanning electron microscopy images of microparticles: (**a**) alginate microparticles containing *L. casei* LC03 (MAL) and (**b**) alginate microparticles containing *L. casei* coated with a second layer of 0.5% of chitosan fungus (MALC).

Surface ultrastructure analysis of the microparticles using SEM is shown in Figure 3. Different magnifications were used due to the difference in size of the microparticles and the need to show a panoramic view. MAL (Figure 3a) exhibited a bacillary morphology with a wrinkled surface, indicative of the alginate’s inherent porosity and flexibility under freeze-drying conditions [30]. In contrast, MALC showed a smoother, more compact surface with a circular morphology (Figure 3b), reflecting the stabilizing effect of the chitosan coating. Studies have shown that the addition of chitosan can reduce porosity, enhance structural stability, and form a dense, uniform layer around alginate particles [17,36]. This configuration not only aids in protecting the encapsulated probiotics but also aligns with the objective of enhancing stability under gastrointestinal and storage conditions [37]. For comparison, spray-dried microcapsules typically result in smaller, less uniform particles (5–150 µm), highlighting the extrusion and ionic gelation techniques as preferable for larger and more stable probiotic encapsulation [28,38].

The application of a fungal chitosan coating (MALC) increased the microparticle size by 51%, attributed to the formation of polyelectrolyte complexes where a thin chitosan layer binds to the alginate matrix. This adjustment did not significantly affect the microparticle size distribution, maintaining a micrometer-scale range. Similar observations were made by Lopes et al. [28] and Apiwattanasiri et al. [22], who reported that chitosan-based coatings enhance the stability and durability of encapsulated materials. In a related study, Thinkohkaew et al. [34] showed that probiotic microencapsulation using alginate and gellan gum with a chitosan coating resulted in a uniform particle distribution, approximately 500–600 μm in diameter. This structure contributed to a denser and more robust microcapsule, highlighting the role of chitosan in improving the mechanical stability and compactness of probiotic microparticles [34].

It is widely known that alginate capsules tend to shrink during the drying phase, often resulting in shape distortion, pore formation, and issues with evaporation, which can compromise stability [17]. Similarly, Zaeim et al. [36] observed that alginate–chitosan microparticles offered a smoother surface structure, attributed to the interaction between chitosan molecules and calcium ions, leading to improved encapsulation integrity and reduced roughness on the particle surface.

This smoother surface is likely due to the competition between chitosan and calcium ions to bind with alginate, enhancing structural compactness and reducing porosity [28]. The addition of a chitosan coating has been shown to enhance the permeability and stability of alginate-based microparticles, resulting in a dense, uniform surface layer that significantly contributes to probiotic protection [38]. Consequently, chitosan effectively covered the porosities within the alginate matrix, which could enhance the overall stability and protection of encapsulated probiotics, potentially improving their viability during gastrointestinal transit and storage [35].

### 3.2. Encapsulation Efficiency (EE) L. casei LC03

The encapsulation efficiency (Table 1) of alginate microparticles containing *L. casei* (MAL) and chitosan-coated alginate microparticles containing *L. casei* (MALC), produced using the ionic gelation technique, was approximately 90.2 ± 0.47% and 90.7 ± 0.58%, respectively. Encapsulation efficiency represents the concentration of viable probiotic cells within the alginate microparticles relative to the initial concentration of free probiotic cells in the suspension used to produce these microparticles (MAL and MALC). Similar reductions in probiotic counts were observed for MAL (8.02 log CFU/mL) and MALC (8.07 log CFU/mL) compared to the free probiotic cell suspension (8.89 log CFU/mL). Therefore, the ionic gelation technique resulted in low probiotic losses during microencapsulation.

The inclusion of polysaccharide compounds, such as chitosan and alginate, in the encapsulation matrix significantly enhances the encapsulation efficiency and stability of probiotic cultures [17,22,34,35,36]. A chitosan coating is associated with high encapsulation efficiency, likely due to the polymer’s ability to form a dense and less porous structure [34,36]. This robust barrier enhances the protection of probiotics during storage and transit through the gastrointestinal tract, ensuring a stable and effective delivery system [17,22,28,37].

In general, successful microencapsulation requires careful consideration of several factors, including the selection of a suitable probiotic strain, an understanding of the stress factors that affect probiotic survival, and the choice of an appropriate hydrogel for encapsulation [17]. Optimizing encapsulation techniques not only improves the stability and delivery of probiotics but also ensures their efficacy as functional ingredients in food products [37].

### 3.3. Viability of Encapsulated L. casei LC03 During Storage

Table 2 presents the viability of free and encapsulated (MAL and MALC) cells of *L. casei* LC03 during 28 days of refrigerated storage at 4 ± 1 °C. Free cells of *L. casei* LC03 showed a significant reduction in viability, with a decrease of 2.153 log CFU/mL after 28 days. In contrast, encapsulated *L. casei* LC03 (MAL and MALC) exhibited greater survival, with reductions of 1.304 and 1.397 log CFU/mL, respectively. The free cell group displayed a significant decrease between days 0–21 (*p* = 0.014) and 0–28 (*p* = 0.02).

The ionic gelation technique effectively encapsulates probiotics within a gel matrix, shielding them from mechanical, thermal, and gastrointestinal stressors. However, during microencapsulation, limitations such as mechanical stress and temperature fluctuations can impact the stability and viability of probiotics in the matrix [17,18]. In the present study, a stable microencapsulation system was achieved using alginate as the primary encapsulating material, which gels in the presence of calcium ions, forming a semi-permeable barrier around the probiotics. The addition of chitosan as a secondary coating further enhances protection; the positively charged chitosan interacts with the negatively charged alginate, creating a denser, less porous structure environment [26,34,36,39,40]. The chitosan coating on the alginate–chitosan microparticle (MALC) provided enhanced protection to probiotics during 28 days of storage, supporting extended viability over longer periods [41]. This aligns with findings showing that chitosan coatings can significantly improve the stability and viability of encapsulated probiotics by forming a protective barrier that resists harsh storage conditions and gastrointestinal environments [39].

### 3.4. Assessment of the Uncoated and Coated Strawberry Samples’ Aging

#### 3.4.1. Physicochemical Changes During Storage of Uncoated Strawberries and Strawberries Coated with the Probiotic Edible Coating

**Figure 4:**
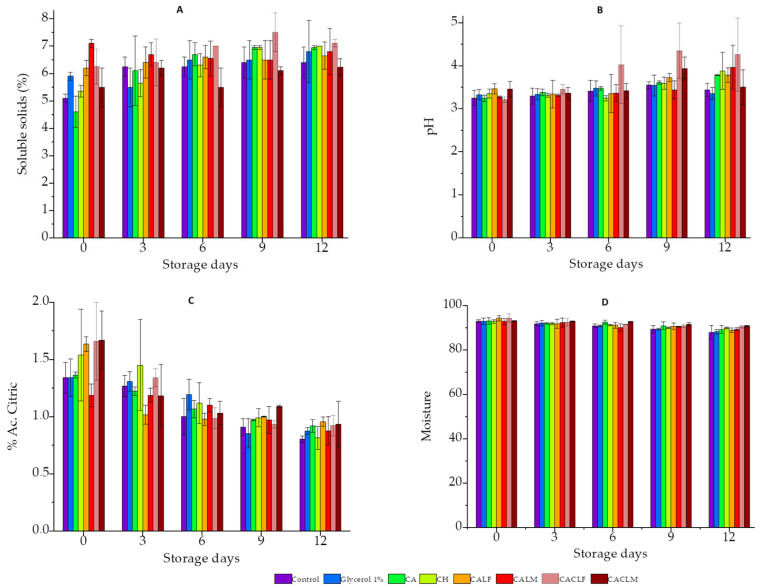
Values of physicochemical parameters of strawberry fruits stored in refrigeration (4 ± 1 °C) for 12 days with the following treatments applied: Control, Glycerol 1% (*v*/*v*), Alginate 3% (CA), Chitosan 0.5% (CH), edible coating with alginate incorporated with *L. case* LCO3 free cells (CALF), edible coating with alginate incorporated with *L. casei* LCO3 microencapsulated (CALM), edible coating with alginate incorporated with *L. casei* LCO3 free cells with a second layer of 0.5% fungal chitosan (CACLF), edible coating with *L. casei* LCO3 microencapsulated in microparticles of alginate with a second layer of 0.5% fungal chitosan (CACLM). (**A**) Soluble solids (%), (**B**) pH, (**C**) titratable acidity (% Ac. Citric), and (**D**) moisture.

Figure 4 shows the physicochemical properties of coated and uncoated strawberry fruits during refrigerated storage (4 ± 1 °C) (Table S1). A significant decrease in soluble solids was observed in the chitosan-coated (CH) group compared to control, glycerol 1%, CA, CALF, CALM, CACLF, and CACLM up to day 6 of storage (Figure 4A). This reduced increase in soluble solids in CH fruits can be attributed to the prevention of innate polysaccharide hydrolysis, as reported in previous studies [31,35].

Most groups showed a significant increase in soluble solids content from day 3 of storage. Similar results were found in Araújo et al. (2024) and Melo et al. (2020) [5,15], where an increase in soluble solids was observed during storage in strawberries coated with an edible chitosan-based coating [5,35]. Soluble solids represent the total amount of soluble sugars, amino acids, and pectin. Their increase during the post-harvest phase may indicate fruit senescence. During storage, soluble solids content tends to increase due to the hydrolysis of polysaccharides into simple sugars [1,5,31].

pH is a crucial indicator of fruit quality, with an increase typically signaling ripening and oxidation. All groups exhibited a gradual pH reduction, with the CAL group showing a significant decrease (*p* < 0.05) during storage (Figure 4B). This pH change can be attributed to the alginate coating, which, being porous, accelerates fruit oxidation and maturation [42]. Furthermore, during the ripening process, the pH of fruits is reduced during storage due to the production of organic acids, enzymatic decomposition, microbial activity that ferments sugars into acids, and the loss of alkaline compounds [42,43].

Titratable acidity, reflecting organic acid content, is another marker of fruit quality. Uncoated (control) and alginate-coated (CA) fruits experienced a reduction in acidity over time (Figure 4C). This trend is consistent with the pH reduction in these groups. Conversely, chitosan-based coatings slowed down the reduction of organic acids, likely due to reduced respiration rates [42,43].

Probiotic-containing groups (CALF, CALM, CACLF, CACLM) did not exhibit significant pH or acidity changes (Figure 4B,C). Although *L. casei* can produce acidic metabolites, no significant pH alteration was observed in strawberries over 12 days of storage. This finding aligns with Wong et al. [44], who observed similar results with carboxymethylcellulose-based probiotic coatings on minimally processed apples.

Regarding moisture content (Figure 4D), the control and 1% glycerol groups experienced higher rates of moisture loss during storage, significantly differing from coated strawberries. Notably, the CALF and CACLM groups exhibited higher moisture values at the end of the experiment (day 12). The semi-permeable nature of the coatings likely reduced water migration to the environment [10,38].

#### 3.4.2. Color Evolution During Cold Storage in Strawberries with Probiotic Edible Coating

**Figure 5:**
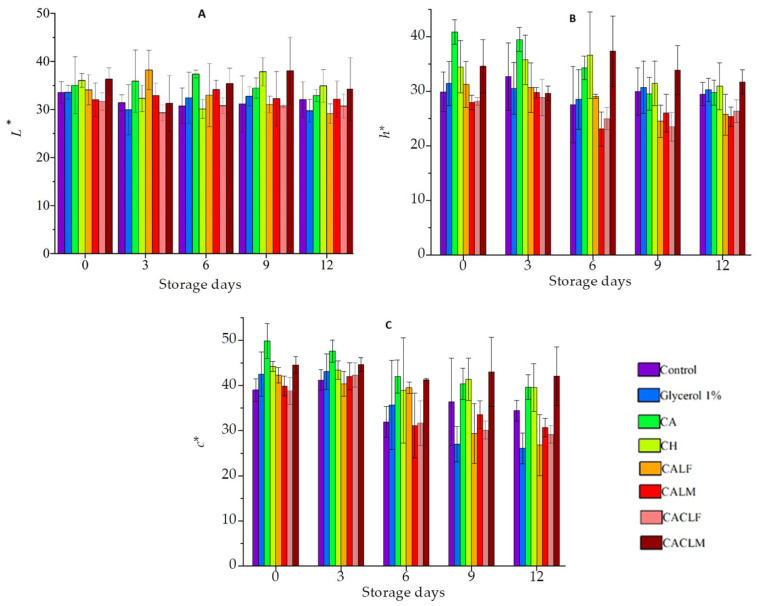
Effect of edible coatings on the color of strawberries during refrigerated storage (4 ± 1 °C) on days 0 to 12 with the following treatments applied: Control, Glycerol 1% (*v*/*v*), Alginate 3% (CA), Chitosan 0.5% (CH), edible coating with alginate incorporated with *L. casei* LCO3 free cells (CALF), edible coating with alginate incorporated with *L. casei* LCO3 microencapsulated (CALM), edible coating with alginate incorporated with *L. casei* LCO3 free cells with a second layer of 0.5% fungal chitosan (CACLF), edible coating with *L. casei* LCO3 microencapsulated in microparticles of alginate with a second layer of 0.5% fungal chitosan (CACLM). (**A**): Luminosity (L*), (**B**): hue angle (h*), (**C**): Chroma (*c).

Color is a key attribute in perceiving strawberry quality, and sensory quality directly influences consumer purchasing decisions. Thus, it is crucial to maintain the fruit’s original characteristics during processing [23]. Figure 5 presents the color results of coated and uncoated strawberry fruits at the beginning of the experiment and after 12 days of refrigerated storage (Table S2). Luminosity (L*) is an indicator of fruit browning. In this study, it was observed that most groups did not exhibit significant variations in L* (*p* > 0.05). However, the CALF group exhibited significant reductions in L* value from day 3 onward, suggesting a decrease in color intensity during storage (Figure 5A). Similar findings were reported by Khodaei and Hamidi-Esfahani [19] and Khodaei and Hamidi-Esfahani [2]. These studies indicate that an edible carboxymethylcellulose coating did not significantly affect the L* value of strawberries.

According to the CIELAB system, the hue angle (h*) indicates color intensity. A lower angle, closer to 0°, corresponds to a more intense red color, while values closer to 90° indicate a more yellow color. Therefore, a decrease in this parameter during storage suggests an intensification of the fruit’s red hue, which is associated with the ripening process and the continued biosynthesis of anthocyanins.

The CH group exhibited the lowest h* values, with statistically significant differences (*p* < 0.05) observed from day 3 onwards (Figure 5B). This indicates that this group had a more vivid core throughout the experiment. These findings align with previous studies using chitosan as an edible coating on strawberries [5,7]. Conversely, the CA group showed higher h* values (*p* < 0.05) compared to control, glycerol 1%, CA, CALF, CALM, CACLF, and CACLM up to day 6 of storage, with an increase in h* from day 3 onwards.

Chroma (C*) is an index that measures color saturation or intensity. Higher values indicate that samples are brighter. The control groups (control and 1% glycerol), CA, CALF, and CACLF showed a reduction in these parameter values during storage (*p* < 0.05), indicating that these strawberries lost their brightness (Figure 5C).

A study on minimally processed apples with an edible probiotic coating observed changes in fruit color with probiotic addition [40]. This reduction in brightness may be associated with probiotic addition, as changes in odor and flavor can be caused by metabolic products of lactic acid bacteria [20,40]

#### 3.4.3. Weight Loss of Strawberries During Cold Storage

**Figure 6:**
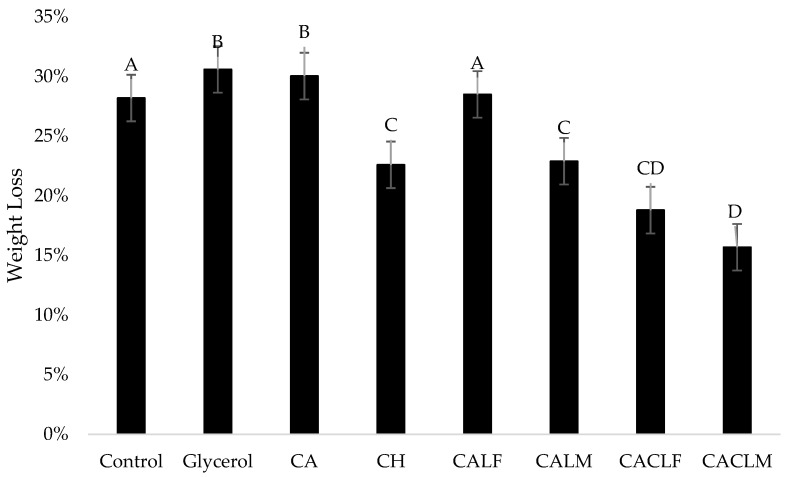
Percentage weight loss of strawberries between the initial and final day (12 days) of refrigerated storage (4 ± 1 °C) with the following treatments applied: control, 1% (*v*/*v*) glycerol, 3% alginate (CA), 0.5% chitosan (CH), alginate edible coating with free *L. casei* LCO3 cells (CALF), alginate edible coating with microencapsulated *L. casei* LCO3 (CALM), edible coating with alginate incorporated with *L. casei* LCO3 free cells with a second layer of 0.5% fungal chitosan (CACLF), edible coating with *L. casei* LCO3 microencapsulated in microparticles of alginate with a second layer of 0.5% fungal chitosan (CACLM). A–D Different superscript capital letters in the same column for the same storage time interval denote differences between the samples (*p* ≤ 0.05), based on the Tukey test.

Weight loss assessment is a widely used indicator for maintaining fruit quality, primarily due to its simplicity [7]. In strawberries, weight loss primarily occurs because of increased respiration and transpiration during post-harvest storage, leading to increased oxidation, moisture loss, and depletion of the fruit’s energy reserves [7,23,44]. Figure 6 illustrates the weight loss of coated and control strawberries during storage. The glycerol 1% and CA groups exhibited the highest percentages of weight loss, showing statistically significant differences compared to the other groups (*p* < 0.05). Statistically significant differences were also observed between the control group and the CH, CALM, CACLF, and CACLM groups, indicating that the groups treated with chitosan coatings demonstrated greater conservation of weight loss when compared with the uncoated group, glycerol 1%, CA, and CALF.

The CACLM group had the lowest percentage of weight loss and was statistically different from the other groups, demonstrating better preservation over the 12-day storage period. Weight loss exceeding 6% can significantly impact the quality of strawberries [7,45]. In this study, the chitosan coating was effective in preserving fruit weight, resulting in weight loss below 6%. Chitosan can act as a barrier, reducing water loss from the fruit [45]. As in studies conducted with papaya [46], pear [15], and guava [43], the chitosan coating helped mitigate weight loss. Added to this, edible coatings can reduce damage to the fruit’s cellular tissues, reducing membrane degradation and liquid leakage and preserving fruit consistency, reducing pathogen growth, so as to extend shelf life and quality during storage [31].

### 3.5. Microbiological Analysis: Fruit Microbiological Quality and Probiotic Viability

The strawberries utilized in this study were sourced from sustainable family farms, ensuring pesticide-free cultivation. Their microbiological quality was evaluated in accordance with Normative Instruction (NI) No. 161 of 1 July 2022, issued by ANVISA, Brazil [47]. This NI mandates the following microbiological safety criteria for whole, fresh fruits: absence of *Salmonella* sp. in 25 g and *E. coli* levels below 10^3^ CFU/g.

To further assess food safety, the presence of coagulase-positive *Staphylococcus* was quantified, with acceptable levels below 10^3^ CFU/g. Additionally, total aerobic mesophilic bacteria and total molds and yeasts were enumerated, as these are common spoilage microorganisms in strawberries, with acceptable levels below 10^6^ CFU/mL [6,8].

Yeast and mold growth in fresh produce is a significant concern, as these microorganisms can significantly degrade product quality. Their enzymes accelerate spoilage, leading to undesirable changes in taste, texture, and appearance. To maintain optimal quality, fresh fruit should ideally contain no more than 10^6^ CFU/g of yeast and mold, as recommended by IFST [3].

**Figure 7:**
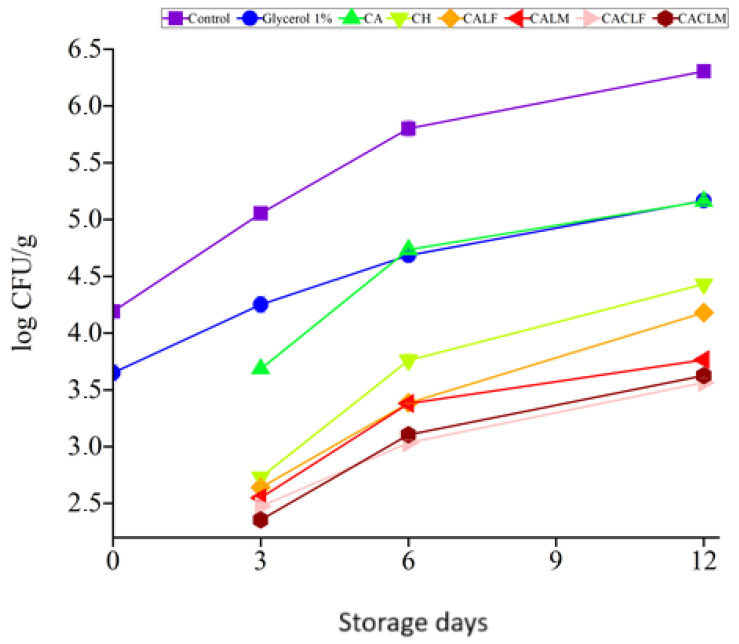
Total aerobic mesophilic bacteria (log CFU/g) of strawberries with different treatments during storage at 4 ± 1 °C for 12 days. Treatments applied were as follows: control, glycerol 1% (*v*/*v*), alginate 3% (CA), chitosan 0.5% (CH), edible coating with alginate incorporated with *L. casei* LCO3 free cells (CALF), edible coating with alginate incorporated with *L. casei* LCO3 microencapsulated (CALM), edible coating with alginate incorporated with *L. casei* LCO3 free cells with a second layer of 0.5% fungal chitosan (CACLF), edible coating with *L. casei* LCO3 microencapsulated in microparticles of alginate with a second layer of 0.5% fungal chitosan (CACLM).

**Figure 8:**
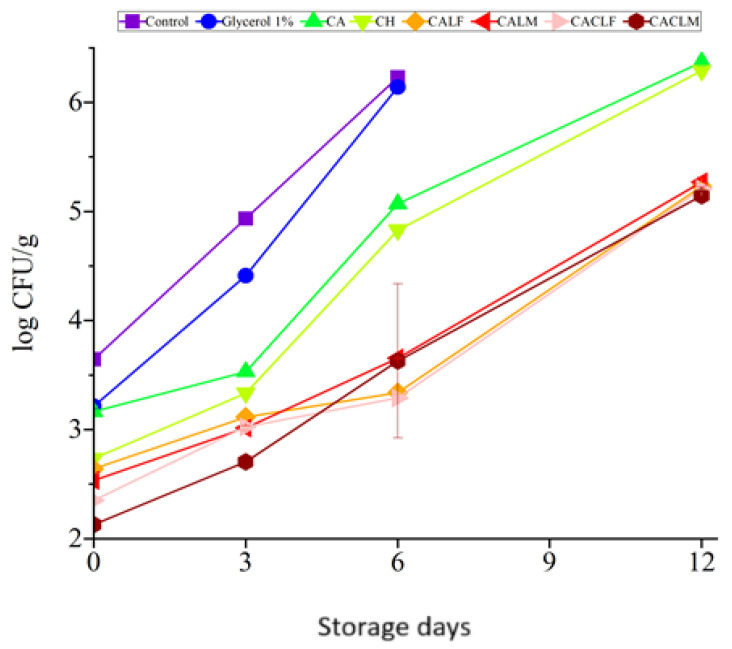
Total yeast and mold count (log CFU/g) of strawberries with different treatments during storage at 4 ± 1 °C for 12 days. Treatments applied were as follows: control, glycerol 1% (*v*/*v*), alginate 3% (CA), chitosan 0.5% (CH), edible coating with alginate incorporated with *L. casei* LCO3 free cells (CALF), edible coating with alginate incorporated with *L. casei* LCO3 microencapsulated (CALM), edible coating with alginate incorporated with *L. casei* LCO3 free cells with a second layer of 0.5% fungal chitosan (CACLF), edible coating with *L. casei* LCO3 microencapsulated in microparticles of alginate with a second layer of 0.5% fungal chitosan (CACLM).

The strawberries, regardless of treatment, adhered to Normative Instruction No. 161 throughout the 12-day storage period at 4 ± 1 °C. No *Salmonella* sp. was detected, and *E. coli*/total coliforms and coagulase-positive *Staphylococcus* were below 10^3^ CFU/g. However, significant differences emerged among treatments in terms of total aerobic mesophilic bacteria (Figure 7, Table S3) and total mold/yeast counts (Figure 8).

Microbiologically, the addition of *L. casei* significantly reduced (*p* ≤ 0.05) the populations of total aerobic mesophilic bacteria and yeasts and molds. Coatings containing the alginate *L. casei* with a chitosan bilayer (CACLF, CACLM) exhibited the lowest microbial counts, followed by the alginate *L. casei* coatings (CALF, CALM), and all were below the maximum acceptable levels of 10^5^ CFU/g for total aerobic mesophilic bacteria and 10^6^ CFU/g for yeasts, and molds. No significant difference was observed between CACLF and CACLM (*p* > 0.05).

Coatings with only alginate and chitosan were less effective, with final yeast and mold counts exceeding 10^6^ CFU/g (*p* ≤ 0.05). In contrast, CACLF and CACLM coatings-maintained yeast and mold counts below this threshold, demonstrating their superior ability to inhibit microbial growth.

The results of the percentage of fruits unfit for consumption (Figure 1 and Figure 8) corroborate the observed fungal growth behavior (Table S4). While control strawberries exhibited visible spoilage as early as day 6, the first signs of spoilage were delayed until day 9 for CH and CALF, and day 12 for CALM, CACLF, and CACLM. Although the latter treatments displayed significant fungal growth at later stages, they maintained lower proliferation rates, especially toward the end of storage, demonstrating the effectiveness of the coatings in inhibiting fungal growth in strawberries.

The percentage of unfit fruits correlated not only with fungal growth but also with other senescence-related parameters, such as mass loss. This multifactorial impact on strawberry quality loss underscores the efficacy of the applied coatings in delaying these processes [3]. Our findings align with those of [2], who also reported lower levels of aerobic mesophilic bacteria, molds, and yeasts in strawberries (*Fragaria × ananassa*) coated with *Lactobacillus rhamnosus* and inulin-enriched gelatin films.

In contrast to our findings, Bambace et al. [20] reported increased aerobic mesophilic bacteria in blueberries coated with *Lactobacillus rhamnosus* CECT 8361 and inulin/oligofructose in alginate. However, they did not observe significant differences in molds and yeasts. This discrepancy may be attributed to the different probiotic strains used. While *L. rhamnosus* supports aerobic conditions, *L. casei*, employed in our study, is preferably anaerobic.

Oliveira et al. [10] reported that probiotics can inhibit the growth of pathogenic and spoilage microorganisms on fruits and vegetables. This antimicrobial effect is likely due to the production of bacteriocins, organic acids, hydrogen peroxide, and diacetyl, as well as competition for nutrients. Incorporating probiotics or their metabolites into edible coatings is a promising strategy to enhance food safety and extend shelf life. However, it is important to consider the encapsulation material of the probiotic, as this can impact on the permeability of the metabolites produced by the probiotic, causing variations in the antimicrobial activity, as well as in the capacity of the microcapsule structure to protect the viability of the probiotic.

Chitosan, a natural polymer, is known for its biocompatibility, biodegradability, and antimicrobial properties. It can be used to create films or coatings that reduce respiration rates and control microbial growth [44]. The acidic nature of chitosan solutions can further contribute to microbial inhibition [12]. These properties make chitosan a valuable tool for improving the shelf life and safety of fruits and vegetables.

Our research highlights the potential of *L. casei* and chitosan coatings to significantly improve the microbiological safety of strawberries.

**Figure 9:**
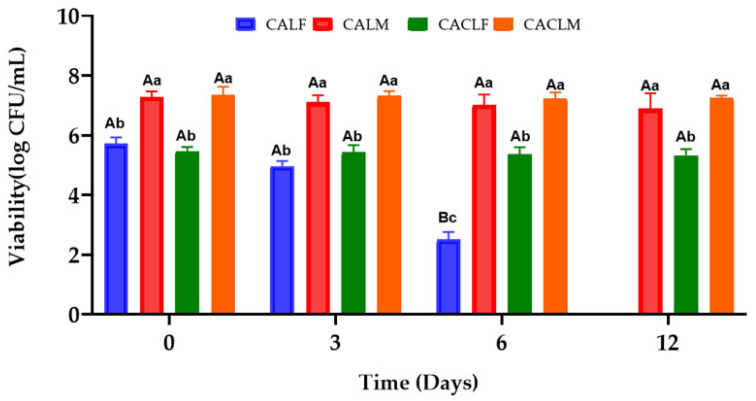
Survival of *L. casei* LC03 (log CFU/g) in strawberries with different treatments during storage at 4 ± 1 °C for 12 days. Treatments applied were as follows: edible coating with alginate incorporated with *L. casei* LCO3 free cells (CALF), edible coating with alginate incorporated with *L. casei* LCO3 microencapsulated (CALM), edible coating with alginate incorporated with *L. casei* LCO3 free cells with a second layer of 0.5% fungal chitosan (CACLF), edible coating with *L. casei* LCO3 microencapsulated in microparticles of alginate with a second layer of 0.5% fungal chitosan (CACLM). A,B Different superscript capital letters in the same row for the same sample at different storage time intervals denote difference (*p* ≤ 0.05), based on the Tukey test. a–c Different superscript small letters in the same column for the same storage time interval denote differences between the samples (*p* ≤ 0.05), based on the Tukey test.

Figure 9 presents the survival rates of *L. casei* in the four treatments during cold storage (Table S5). Notably, free-form *L. casei* (CALF and CACLF) exhibited the lowest viability, especially CALF, which achieved zero survival at the end of the study. Although the addition of chitosan (CACLF) offered some protection, reducing the loss of viability to just 0.14 log CFU/mL, this was not statistically significant (*p* > 0.05).

In contrast, encapsulated *L. casei* (CALM and CACLM) maintained viability throughout storage (*p* > 0.05), highlighting the protective effect of encapsulation, which allows the retention of the compound within the particles and its controlled release [28].

Interestingly, the inclusion of chitosan in the encapsulation matrix (CACLM) did not enhance probiotic survival compared to the coating without chitosan (CALM). However, when differences were observed between CALM and CACLF (chitosan-coated free probiotic), probiotic survival was significantly higher in the edible coating containing chitosan. This suggests that chitosan may offer protective effects to the free probiotic strain [28].

Furthermore, although used at a sub-MIC concentration for *L. casei*, chitosan at 0.5% (5 mg/mL) exhibits antimicrobial activity against various microorganisms. This indicates that, while not directly inhibiting the probiotic, chitosan could potentially enhance probiotic survival by inhibiting competing food spoilage microorganisms on the strawberry’s surface.

At the end of the 12-day period, only encapsulated treatments retained viable cell counts above 10^6^–10^7^ CFU/mL, a level sufficient for potential health benefits [3].

### 3.6. Total Phenolic Content (TPC)

Table 3 presents the total phenolic content (TPC) of uncoated and differently coated strawberries during 12 days of refrigerated storage at 4 °C. A significant decrease in TPC (*p* ≤ 0.05) was observed across all groups, irrespective of treatment, over the storage period. The most substantial TPC reductions (*p* ≤ 0.05) were noted in control (uncoated) strawberries, those coated solely with 1% glycerol, and those coated solely with 3% alginate, exhibiting a 50–44% decline compared to initial values. Conversely, the remaining groups (CH, CALF, CALM, CACLF, and CACLM) experienced TPC reductions below 40% (*p* ≤ 0.05). Notably, the CACLM, CACLF, and CALM groups maintained TPC without significant difference (*p* ≤ 0.05) until the third day of storage. However, from the sixth day onward, they displayed a significant TPC reduction (*p* ≤ 0.05) until the end of the storage period. In contrast, the control, 1% glycerol, CA, CH, and CALF groups exhibited earlier significant TPC reductions, commencing on the third day and persisting until the end of storage. As shown in Table 3, the 0.5% fungal chitosan bilayer, particularly in the group containing *L. casei* microencapsulated in alginate capsules (CACLM), contributed to delaying TPC loss in strawberries.

These findings align with the observations of Chikhala et al. [23], who investigated the impact of xanthan gum coatings enriched with *Lactiplantibacillus plantarum* 75 or *Bifidobacterium longum* on the post-harvest quality of fresh-cut Honeydew (*Cucumis melo* L.) and cantaloupe (*Cucumis melo* var. cantalupensis) melons stored at 5 °C for 5 days. They reported that probiotic-infused coatings mitigated the loss of total phenolic compounds compared to uncoated fruits. The authors proposed that the probiotic cells forming a protective layer on the fresh-cut melon surface impeded oxygen diffusion and enzymatic oxidation of polyphenols.

Additionally, the incorporation of a 0.5% fungal chitosan layer could have contributed to the improved retention of total phenolic compounds in strawberries (CACLF and CACLM). Chitosan is a natural polymer that has been shown to delay the ripening process by reducing respiration rate and metabolic activity in fruits [6]. This effect is independent of the presence of other bioactive compounds, such as essential oils or probiotic cells, suggesting that chitosan’s ability to maintain fruit quality is intrinsic to its properties.

## 4. Conclusions

The *L. casei* LC03 alginate microcapsules showed uniform morphology and reduced porosity when added to the fungal chitosan layer, different from the MAL group, in which it was possible to observe wrinkling visualized in scanning electron microscopy. It is possible to verify that the fungal chitosan guaranteed protection against stressors during the processing of microcapsules. The double-layer ionic gelation (MALC) technology did not bring significant losses when compared to the control groups, making it viable for use as a protective technology for probiotics. On the other hand, free cells suffered significant loss during storage, which impairs their intake in food, since processing is responsible for reducing probiotic reduction.

The probiotic edible coating contributed to the conservation of strawberry quality characteristics, maintaining pH, preserving titratable acidity, and reducing moisture loss during drying. For color parameters, strawberries that presented the probiotic in its free form had reduced fruit brightness. Edible coatings with chitosan promoted a significant reduction in weight loss during the storage period in strawberries, reducing fruit deterioration. These results contribute to expanding knowledge of the addition of probiotics to edible coatings for minimally processed tropical fruits, and contribute to a product with convenience, practicality, and quality parameters maintained throughout refrigerated storage, suggesting that strawberries can be offered as a functional probiotic food.

## Figures and Tables

**Table 1:** Encapsulation efficiency (EE%); average ± standard deviation; n = 3 of *L. casei* LC03 (log CFU/mL) before (free form) and after microencapsulation in microparticles of alginate (MAL) and in microparticles of alginate coated with a second layer of 0.5% fungal chitosan (MALC).

Viable Cell Count (log CFU/mL)			
Microparticle Type	Number of Initial Cells	Number of Microencapsulated Cells	EE%
MAL	8.89 ±0.41 ^a^	8.02 ±0.28 ^a^	90.2 ± 0.47 ^a^
MALC	8.89 ±0.41 ^a^	8.07 ±0.21 ^a^	90.7 ± 0.58 ^a^

^a^: equal superscript letters in the same column do not denote significant differences (*p* > 0.05) in microencapsulation yield in different types of microparticles, according to Tukey test.

**Table 2:** Survival rate (log UFC/mL) (average ± standard deviation; n = 3) of *L. casei* LC03 free, *L. casei* LC03 encapsulated in microparticles of alginate (MAL), and *L. casei* LC03 encapsulated in microparticles of alginate with a second layer of 0.5% fungal chitosan (MALC) in water distilled during 28 days of refrigeration storage (4 ± 1 °C).

Time (Days)					
Samples	0	7	14	21	28
Free cells	9.023 ± 0.62 ^a^	8.707 ± 0.52 ^ab^	7.780 ± 1.68 ^b^	7.033 ± 1.16 ^bc^	6.870 ± 1.18 ^c^
MAL	8.917 ± 0.74 ^a^	8.680 ± 0.96 ^a^	8.297 ± 0.55 ^a^	7.877 ± 0.78 ^a^	7.613 ± 1.78 ^a^
MALC	8.770 ± 1.75 ^a^	8.473 ± 1.89 ^a^	8.073 ± 2.38 ^a^	7.620 ± 2.65 ^a^	7.373 ± 1.26 ^a^

^a–c^ Different superscript small letters in the same row for the same sample at different storage time intervals denote difference (*p* ≤ 0.05), based on the Tukey test.

**Table 3:** Total phenolic content (mg GAE/kg) of strawberries at days 0, 3, 6, and 12 during refrigerated storage (4 ± 1 °C) with the following treatments applied: control, glycerol 1% (*v*/*v*), alginate 3% (CA), chitosan 0.5% (CH), edible coating with alginate incorporated with *L. casei* LCO3 free cells (CALF), edible coating with alginate incorporated with *L. casei* LCO3 microencapsulated (CALM), edible coating with alginate incorporated with *L. casei* LCO3 free cells with a second layer of 0.5% fungal chitosan (CACLF), edible coating with *L. casei* LCO3 microencapsulated in microparticles of alginate with a second layer of 0.5% fungal chitosan (CACLM).

Time (Days)				
Samples	0	3	6	12
Control	2684.45 (±6.29) ^Af^	2450.95 (±11.10) ^Be^	1926.57 (±2.34) ^Cbc^	1485.50 (±3.54) ^Dg^
Glycerol 1%	2704.41 (±0.70) ^Ae^	2532.11 (±1.68) ^Bd^	1781.05 (±4.19) ^Cc^	1366.37 (±3.49) ^Dh^
CA	2883.00 (±4.24) ^Ad^	2606.04 (±3.73) ^Bc^	2038.90 (±1.27) ^Cb^	1573.55 (±0.49) ^Df^
CH	2995.50 (±3.54) ^Aa^	2766.30 (±3.82) ^Bb^	2336.09 (±3.10) ^Ca^	1855.90 (±2.84) ^De^
CALF	2926.41 (±3.53) ^Ac^	2768.91 (±0.55) ^Bb^	2366.41 (±3.53) ^Ca^	1916.49 (±3.27) ^Dd^
CALM	2965.32 (±2.14) ^Ab^	2776.41 (±3.54) ^ABb^	2506.00 (±174.37) ^Ba^	1975.35 (±2.18) ^Cc^
CACLF	2992.97 (±2.74) ^Aa^	2973.34 (±0.79) ^Aa^	2419.92 (±0.73) ^Ba^	2015.95 (±24.13) ^Cb^
CACLM	3001.88 (±4.19) ^Aa^	2987.66 (±2.32) ^Aa^	2494.98 (±1.53) ^Ba^	2092.52 (±8.89) ^Ca^

A–D Different superscript capital letters in the same row for the same sample at different storage time intervals denote difference (*p* ≤ 0.05), based on the Tukey test. a–h Different superscript small letters in the same column for the same storage time interval denote differences between the samples (*p* ≤ 0.05), based on the Tukey test.

## Data Availability

The original contributions presented in this study are included in the article. Further inquiries can be directed to the corresponding author.

## Supplementary Materials

The following supporting information can be downloaded at: https://www.mdpi.com/article/10.3390/foods14020203/s1, Table S1. Values of physicochemical parameters of strawberry fruits stored at refrigeration (4 ± 1 °C) for 12 days; Table S2. Effect of edible coatings on the color of strawberries during refrigerated storage (4 ± 1°C) on days 0 to 12; Table S3. Total aerobic mesophilic bacteria (log CFU/g) of strawberries with different treatments during storage at 4 ± 1 °C for 12 days; Table S4. Total yeast and mold count (log CFU/g) of strawberries with different treatments during storage at 4 ± 1 °C for 12 days; Table S5. Survival of *L. casei* LC03 (log CFU/g) in strawberries with different treatments during storage at 4 ± 1 °C for 12 days.
