# Supplementary material for: Edible Alginate–Fungal Chitosan Coatings as Carriers for Lacticaseibacillus casei LC03 and Their Impact on Quality Parameters of Strawberries During Cold Storage
Source: Foods. 2025 Jan 10;14(2):203. doi: 10.3390/foods14020203 (PMC11765008; doi:10.3390/foods14020203)
Supplement: Supplementary file 1 [file foods-14-00203-s001.zip › foods-3388294-supplementary.pdf]

Table S1. Values of physicochemical parameters of strawberry fruits stored at refrigeration (4 ± 1 °C) for 12 days with the following treatments applied: Control, Glycerol 1% (*v*/*v*), Alginate 3% (CA), Chitosan 0,5% (CH), edible coating with alginate incorporated with *L. casei* LCO3 free cells (CALF), edible coating with alginate incorporated with *L. casei* LCO3 microencapsulated (CALM), edible coating alginate incorporated with *L. casei* LCO3 free cells with a second layer of 0.5% fungal chitosan (CACLF), edible coating with *L. casei* LCO3 microencapsulated in microparticles of alginate with a second layer of 0.5% fungal chitosan (CACLM).

Treatments	Storage Days				
	**0**	**3**	**6**	**9**	**12**
**Soluble solids (%)**					
Control	5.10 (±0.14)^{Ab}	6.25 (±0.35)^{Aa}	6.25 (±0.35)^{Aa}	6.40 (±0.57)^{Aa}	6.40 (±0.57)^{Aa}
Glycerol 1%	5.90 (±0.14)^{Aab}	5.50 (±0.71)^{Aa}	6.50 (±0.71)^{Aa}	6.50 (±0.71)^{Aa}	6.80 (±1.13)^{Aa}
CA	4.60 (±0.57)^{Ab}	6.10 (±1.7)^{Aa}	6.70 (±0.42)^{Aa}	6.95 (±0.07)^{Aa}	6.95 (±0.07)^{Aa}
CH	5.35 (±0.21)^{Bb}	5.65 (±0.49)^{Ba}	6.30 (±0.42)^{ABa}	6.95 (±0.07)^{Aa}	7.00 (±0.00)^{Aa}
CALF	6.20 (±0.28)^{Aab}	6.40 (±0.57)^{Aa}	6.60 (±0.42)^{Aa}	6.50 (±0.71)^{Aa}	6.65 (±0.49)^{Aa}
CALM	7.10 (±0.14)^{Aa}	6.70 (±0.42)^{Aa}	6.55 (±0.64)^{Aa}	6.50 (±0.71)^{Aa}	6.80 (±0.85)^{Aa}
CACLF	6.25 (±0.64)^{Aab}	6.40 (±0.85)^{Aa}	7.00 (±0.00)^{Aa}	7.50 (±0.71)^{Aa}	7.10 (±0.14)^{Aa}
CACLM	5.50 (±0.71)^{Aab}	6.20 (±0.28)^{Aa}	5.50 (±0.71)^{Aa}	6.10 (±0.14)^{Aa}	6.23 (±0.31)^{Aa}
**pH**					
Control	3.45 (±0.18)^{Aa}	3.29 (±0.19)^{Aa}	3.31 (±0.25)^{Aa}	3.22 (±0.08)^{Aa}	3.01 (±0.15)^{Aa}
Glycerol 1%	3.33 (±0.12)^{Aa}	3.34 (±0.13)^{Aa}	3.27 (±0.18)^{Aa}	3.34 (±0.24)^{Aa}	3.25 (±0.14)^{Aa}
CA	3.58 (±0.07)^{Bb}	3.53 (±0.08)^{CDa}	3.22 (±0.04)^{Aa}	3.12 (±0.04)^{ABa}	3.08 (±0.01)^{Aa}
CH	3.45 (±0.11)^{Aa}	3.42 (±0.05)^{Aa}	3.34 (±0.06)^{Aa}	3.29 (±0.15)^{Aa}	3.18 (±0.44)^{Aa}
CALF	3.46 (±0.12)^{Aa}	3.34 (±0.32)^{Aa}	3.35 (±0.44)^{Aa}	3.22 (±0.09)^{Aa}	3.09 (±0.17)^{Aa}
CALM	3.67 (±0.02)^{Ca}	3.41 (±0.03)^{Aa}	3.46 (±0.20)^{Aa}	3.34 (±0.21)^{Aa}	3.27 (±0.51)^{Aa}
CACLF	3.61 (±0.06)^{Aa}	3.45 (±0.11)^{Aa}	3.38 (±0.90)^{Aa}	3.22 (±0.64)^{Aa}	3.15 (±0.85)^{Aa}
CACLM	3.66 (±0.18)^{Aa}	3.46 (±0.14)^{Aa}	3.41 (±0.18)^{Aa}	3.21 (±0.27)^{Aa}	3.16 (±0.41)^{Aa}
**Titratable acidity (% Ac. Citric)**					
Control	1.34 (±0.14)^{Aa}	1.27 (±0.09)^{ABa}	1.00 (±0.16)^{Ba}	0.81 (±0.07)^{BCa}	0.80 (±0.03)^{Ca}
Glycerol 1%	1.34 (±0.16)^{Aa}	1.31 (±0.09)^{ABa}	1.02 (±0.13)^{Ba}	0.86 (±0.13)^{Ba}	0.88 (±0.03)^{ABa}
CA	1.36 (±0.03)^{Aa}	1.22 (±0.04)^{ABa}	1.07 (±0.08)^{Ba}	0.87 (±0.01)^{Ba}	0.82 (±0.06)^{BCa}
CH	1.54 (±0.40)^{Aa}	1.45 (±0.40)^{Aa}	1.12 (±0.18)^{Aa}	0.99 (±0.08)^{Aa}	0.92 (±0.10)^{Aa}
CALF	1.63 (±0.06)^{Aa}	1.12 (±0.08)^{Aa}	1.18 (±0.05)^{Aa}	1.00 (±0.00)^{Aa}	0.96 (±0.04)^{Ba}
CALM	1.19 (±0.10)^{Aa}	1.19 (±0.06)^{Aa}	1.12 (±0.06)^{Aa}	0.97 (±0.12)^{Aa}	0.98 (±0.12)^{Aa}
CACLF	1.66 (±0.34)^{Aa}	1.24 (±0.08)^{Aa}	1.19 (±0.09)^{Aa}	0.96 (±0.03)^{Aa}	0.92 (±0.09)^{Aa}
CACLM	1.67 (±0.26)^{Aa}	1.18 (±0.28)^{Aa}	1.13 (±0.10)^{Aa}	1.09 (±0.01)^{Aa}	0.94 (±0.20)^{Aa}
**Moisture**					
Control	92.93 (±0.66)^{Aa}	91.77 (±0.94)^{ABa}	90.84 (±0.86)^{ABa}	88.29 (±1.64)^{ABa}	87,82 (±3.14)^{Ba}
Glycerol 1%	92.86 (±1.46)^{Aa}	92.04 (±1.15)^{ABa}	90.88 (±0.35)^{ABa}	89.33 (±0.30)^{ABa}	88.12 (±1.10)^{Ba}
CA	93.00 (±1.55)^{Aa}	91.90 (±0.42)^{Aa}	92.49 (±0.88)^{Aa}	90.88 (±1.76)^{Aa}	89.23 (±1.73)^{Aa}
CH	92.75 (±0.78)^{Aa}	91.89 (±0.47)^{Aa}	91.14 (±0.37)^{Aa}	90.95 (±0.29)^{Aa}	89.83 (±0.41)^{Aa}
CALF	94.27 (±1.22)^{Aa}	91.68 (±2.02)^{Aa}	91.12 (±1.39)^{Aa}	90.64 (±1.56)^{Aa}	88.86 (±1.05)^{Aa}
CALM	92.79 (±1.44)^{Aa}	92.30 (±2.01)^{Aa}	90.12 (±1.68)^{Aa}	90.40 (±0.14)^{Aa}	89.34 (±0.78)^{Aa}
CACLF	94.23 (±1.83)^{Aa}	92.42 (±1.72)^{Aa}	91.42 (±0.11)^{Aa}	90.76 (±0.69)^{Aa}	90.41 (±0.55)^{Aa}
CACLM	93.04 (±0.23)^{Aa}	92.92 (±0.18)^{Aa}	92.70 (±0.20)^{Aa}	91.53 (±0.74)^{ABa}	90.74 (±0.27)^{Ba}

a–c Different superscript small letters in the same row for the same sample at different storage time intervals denote difference ($p \leq 0.05$), based on the Tukey test. A–D Different superscript capital letters in the same collum for the same storage time interval denote differences between the samples ($p \leq 0.05$), based on the Tukey test.

Table S2. Effect of edible coatings on the color of strawberries during refrigerated storage (4 ± 1°C) on days 0 to 12 with the following treatments applied: Control, Glycerol 1% (v/v), Alginate 3% (CA), Chitosan 0.5% (CH), edible coating with alginate incorporated with *L. casei* LCO3 free cells (CALF), edible coating with alginate incorporated with *L. casei* LCO3 microencapsulated (CALM edible coating alginate incorporated with *L. casei* LCO3 free cells with a second layer of 0.5% fungal chitosan (CACLF), edible coating with *L. casei* LCO3 microencapsulated in microparticles of alginate with a second layer of 0.5% fungal chitosan (CACLM).

Treatments	Storage Days				
	**0**	**3**	**6**	**9**	**12**
L					
Control	33.55 (±2.24)^{Aa}	31.39 (±1.68)^{Aa}	30.73 (±3.71)^{Aa}	31.13 (±5.83)^{Aa}	32.04 (±3.70)^{Aa}
Glycerol 1%	33.58 (±1.44)^{Aa}	29.95 (±5.21)^{Aa}	32.41 (±5.33)^{Aa}	32.73 (±1.97)^{Aa}	29.74 (±2.33)^{Aa}
CA	35.02 (±5.92)^{Aa}	35.89 (±6.49)^{Aa}	37.37 (±0.79)^{Aa}	34.45 (±2.09)^{Aa}	32.90 (±1.24)^{Aa}
CALF	36.03 (±1.45)^{ABa}	32.31 (±2.75)^{ABa}	30.10 (±1.94)^{Ba}	37.86 (±2.94)^{Aa}	34.91 (±3.38)^{ABa}
CALM	34.09 (±3.15)^{Aa}	38.21 (±4.10)^{Aa}	32.97 (±6.57)^{Aa}	31.03 (±1.72)^{Aa}	29.13 (±2.05)^{Aa}
CH	32.03 (±3.50)^{Aa}	32.89 (±2.59)^{Aa}	34.13 (±1.94)^{Aa}	32.25 (±5.65)^{Aa}	32.12 (±3.78)^{Aa}
CACLF	31.66 (±1.80)^{Aa}	29.31 (±1.67)^{Aa}	30.79 (±1.66)^{Aa}	30.75 (±0.33)^{Aa}	30.73 (±2.51)^{Aa}
CACLM	36.32 (±2.31)^{Aa}	31.24 (±5.83)^{Aa}	35.38 (±3.18)^{Aa}	38.05 (±6.94)^{Aa}	34.21 (±6.56)^{Aa}
h^*_{ab}					
Control	29.88 (±3.62)^{Ab}	32.71 (±6.14)^{Aa}	27.55 (±6.99)^{Aab}	29.94 (±4.35)^{Aa}	29.49 (±2.09)^{Aa}
Glycerol 1%	31.41 (±4.01)^{Aab}	30.54 (±4.78)^{Aa}	28.52 (±5.45)^{Aab}	30.73 (±4.76)^{Aa}	30.27 (±2.12)^{Aa}
CA	35.89 (±2.23)^{Aa}	39.47 (±2.22) ^{Ba}	34.27 (±2.19)^{BCab}	29.54 (±2.98)^{Ca}	29.74 (±2.30)^{Ca}
CALF	34.47 (±4.80)^{Aab}	32.76 (±4.56)^{Aa}	32.60 (±7.89)^{Aa}	31.43 (±4.07)^{Aa}	30.94 (±4.29)^{Aa}
CALM	31.25 (±4.22)^{Aab}	30.68 (±4.54)^{Aa}	29.06 (±0.38)^{Aab}	24.51 (±2.97)^{Aa}	25.74 (±3.75)^{Aa}
CH	28.99 (±1.34)^{Ab}	26.74 (±0.95)^{ABa}	23.08 (±3.15)^{Bb}	26.00 (±3.44)^{ABa}	25.32 (±1.83)^{ABa}
CACLF	28.15 (±0.70)^{Ab}	28.86 (±3.35)^{Aa}	24.95 (±2.03)^{Aab}	23.47 (±2.60)^{Aa}	26.35 (±2.09)^{Aa}
CACLM	34.57 (±4.87)^{Aa}	32.60 (±1.33)^{Aa}	33.33 (±6.49)^{Aa}	33.89 (±4.49)^{Aa}	31.74 (±2.23)^{Aa}
C^*_{ab}					
Control	39.02 (±2.52)^{Ab}	41.23 (±2.32)^{Aa}	35.97 (±3.38)^{Ba}	32.40 (±9.68)^{Ba}	34.42 (±2.3)^{Aa}
Glycerol 1%	42.54 (±4.89)^{Aab}	43.08 (±3.91)^{Aa}	35.69 (±9.87)^{Ba}	26.99 (±3.94)^{Ba}	26.07 (±3.39)^{Ba}
CA	49.85 (±3.91)^{Ab}	47.62 (±2.46)^{ABa}	42.00 (±3.69)^{Aa}	40.39 (±3.44)^{Aa}	38.65 (±2.72)^{Aa}
CALF	44.26 (±1.10)^{Aab}	43.38 (±2.04)^{Aa}	38.93 (±11.67)^{Aa}	41.39 (±4.64)^{Aa}	39.57 (±5.26)^{Aa}
CALM	42.28 (±1.75)^{Aa}	42.40 (±2.77)^{Aa}	39.50 (±1.23)^{Aa}	39.41 (±6.63)^{Aa}	38.79 (±6.79)^{Aa}
CH	39.88 (±2.19)^{Aa}	42.04 (±2.99)^{Aa}	38.13 (±7.19)^{Aa}	38.55 (±3.07)^{ABa}	38.69 (±2.00)^{Aa}
CACLF	38.76 (±2.96)^{Ab}	42.31 (±2.68)^{Aa}	41.66 (±4.95)^{Aa}	30.08 (±1.98)^{Ca}	29.17 (±1.96)^{Ca}
CACLM	44.55 (±1.80)^{Aab}	44,63 (±1,54)^{Aa}	41,30 (±0.25)^{Aa}	43.04 (±7.66)^{Aa}	42.10 (±6.47)^{Aa}

a–c Different superscript small letters in the same row for the same sample at different storage time intervals denote difference ($p \leq 0.05$), based on the Tukey test. A–D Different superscript capital letters in the same collum for the same storage time interval denote differences between the samples ($p \leq 0.05$), based on the Tukey test.

Table S3. Total aerobic mesophilic bacteria (log CFU/g) of strawberries with different treatments during storage at 4 ± 1 °C for 12 days. Treatments applied: Control, Glycerol 1% (v/v), Alginate 3% (CA), Chitosan 0.5% (CH), edible coating with alginate incorporated with *L. casei* LCO3 free cells (CALF), edible coating with alginate incorporated with *L. casei* LCO3 microencapsulated (CALM), edible coating alginate incorporated with *L. casei* LCO3 free cells with a second layer of 0.5% fungal chitosan (CACLF), edible coating with *L. casei* LCO3 microencapsulated in microparticles of alginate with a second layer of 0.5% fungal chitosan (CACLM).

	Time (days)			
**Samples**	**0**	**3**	**6**	**12**
Control	4.19(±0.06)^{D}	5.06(±0.02)^{Ca}	5.80(±0.07)^{Ba}	6.31(±0.01)^{Aa}
Glycerol 1%	3.65(±0.07)^{D}	4.25(±0.07)^{Cb}	4.69(±0.04)^{Bb}	5.17(±0.04)^{Ab}
CA	Ab	3.69(±0.01)^{Cc}	4.74(±0.04)^{Bb}	5.16(±0.01)^{Ab}
CH	Ab	2.73(±0.03)^{Cd}	3.76(±0.06)^{Bc}	4.43(±0.04)^{Ac}
CALF	Ab	2.64(±0.01)^{Cde}	3.39(±0.01)^{Bd}	4.18(±0.00)^{Ad}
CALM	Ab	2.55(±0.03)^{Cef}	3.38(±0.01)^{Bd}	3.77(±0.02)^{Ae}
CACLF	Ab	2.48(±0.01)^{Cfg}	3.04(±0.02)^{Be}	3.57(±0.04)^{Af}
CACLM	Ab	2.36(±0.04)^{Cg}	3.11(±0.02)^{Be}	3.63(±0.02)^{Af}

A–D Different superscript capital letters in the same row for the same sample at different storage time intervals denote difference ($p \leq 0.05$), based on the Tukey test. a–g Different superscript small letters in the same collum for the same storage time interval denote differences between the samples ($p \leq 0.05$), based on the Tukey test. Ab-Absent

Table S4. Total yeast and mold count (log CFU/g) of strawberries with different treatments during storage at 4 ± 1 °C for 12 days. Treatments applied: Control, Glycerol 1% (v/v), Alginate 3% (CA), Chitosan 0,5% (CH), edible coating with alginate incorporated with *L. casei* LCO3 free cells (CALF), edible coating with alginate incorporated with *L. casei* LCO3 microencapsulated (CALM), edible coating alginate incorporated with *L. casei* LCO3 free cells with a second layer of 0.5% fungal chitosan (CACLF), edible coating with *L. casei* LCO3 microencapsulated in microparticles of alginate with a second layer of 0.5% fungal chitosan (CACLM).

	Time (days)			
**Samples**	**0**	**3**	**6**	**12**
Control	3.65(±0.02)^{Ca}	4.94(±0.04)^{Ba}	6.23(±0.01)^{Aa}	UN
Glycerol 1%	3.22(±0.02)^{Cb}	4.41(±0.01)^{Bb}	6.14(±0.01)^{Aa}	UN
CA	3.17(±0.01)^{Db}	3.53(±0.03)^{Cc}	5.07(±0.01)^{Bb}	6.37(±0.04)^{Aa}
CH	2.74(±0.04)^{Dc}	3.34(±0.04)^{Cd}	4.83(±0.01)^{Bb}	6.29(±0.01)^{Ab}
CALF	2.64(±0.04)^{Dcd}	3.12(±0.02)^{Ce}	3.34(±0.01)^{Bc}	5.23(±0.03)^{Ad}
CALM	2.54(±0.04)^{Dd}	3.02(±0.02)^{Ce}	3.66(±0.01)^{Bc}	5.27(±0.04)^{Ac}
CACLF	2.35(±0.00)^{De}	3.03(±0.04)^{Ce}	3.29(±0.01)^{Bc}	5.21(±0.01)^{Ae}
CACLM	2.13(±0.04)^{Cf}	2.71(±0.01)^{BCf}	3.63(±0.71)^{Bc}	5.15(±0.04)^{Af}

A–D Different superscript capital letters in the same row for the same sample at different storage time intervals denote difference ($p \leq 0.05$), based on the Tukey test. a–f Different superscript small letters in the same collum for the same storage time interval denote differences between the samples ($p \leq 0.05$), based on the Tukey test. UN (uncountable)- count above 7 Log CFU/g,

Table S5. Survival of *L. casei* LC03 (log CFU/g) in strawberries with different treatments during storage at 4±1 ºC for 12 days. Treatments applied: edible coating with alginate incorporated with *L. casei* LCO3 free cells (CALF), edible coating with alginate incorporated with *L. casei* LCO3 microencapsulated (CALM), edible coating alginate incorporated with *L. casei* LCO3 free cells with a second layer of 0.5% fungal chitosan (CACLF), edible coating with *L. casei* LCO3 microencapsulated in microparticles of alginate with a second layer of 0.5% fungal chitosan (CACLM).

	Time (days)			
**Samples**	**0**	**3**	**6**	**12**
**CALF**	5.72(±0.21)^{Ab}	4.97(±0.17)^{Ab}	2.51(±0.25)^{Bc}	0.00(±0.00)^{Cc}
**CALM**	7.29(±0.18)^{Aa}	7.10(±0.24)^{Aa}	7.02(±0.35)^{Aa}	6.89(±0.52)^{Aa}
**CACLF**	5.47(±0.14)^{Ab}	5.43(±0.24)^{Ab}	5.37(±0.23)^{Ab}	5.33(±0.21)^{Ab}
**CACLM**	7.36(±0.27)^{Aa}	7.32(±0.16)^{Aa}	7.24(±0.20)^{Aa}	7.25(±0.08)^{Aa}

A–C Different superscript capital letters in the same row for the same sample at different storage time intervals denote difference ($p \leq 0.05$), based on the Tukey test. a–c Different superscript small letters in the same collum for the same storage time interval denote differences between the samples ($p \leq 0.05$), based on the Tukey test.
